# Variant Distal Ulnar Nerve Loop: A Previously Undescribed Anatomical Finding

**DOI:** 10.7759/cureus.2604

**Published:** 2018-05-10

**Authors:** Mayank Patel, Joe Iwanaga, Rod J Oskouian, R. Shane Tubbs

**Affiliations:** 1 Clinical Anatomy Research, Seattle Science Foundation, Seattle, USA; 2 Seattle Science Foundation, Seattle, USA; 3 Neurosurgery, Swedish Neuroscience Institute, Seattle, USA; 4 Neurosurgery, Seattle Science Foundation, Seattle, USA

**Keywords:** ulnar nerve, loop, variations, anatomy, anomalies, cadaveric, hand surgery

## Abstract

A previously undescribed variant ulnar nerve loop was discovered during the routine anatomic forearm and hand dissection of an adult female. The major finding was that of a large loop traveling around the distal tendon of the flexor carpi ulnaris. The variation presented here appears to be unique. The exact function of such derailed anatomy is not clear but, if found during surgery, might confound normal dissection methods or, when present, could result in varied clinical presentations regarding the sensory or motor examination of the hand.

## Introduction

Variations are commonly seen involving the nerves of the forearm and hand. Ulnar nerve injury is the most common peripheral nerve injury of the upper extremity requiring hospitalization. Therefore, understanding the variations of this nerve can provide better outcomes with surgical treatments or assist in deciphering unusual physical examinations. 

After originating from the medial cord of the brachial plexus, the ulnar nerve courses along the medial arm to pass behind the medial epicondyle and then enters the forearm between the two heads of the flexor carpi ulnaris muscle. It travels distally in the forearm along the anterior border of flexor digitorum profundus. Proximal to the wrist, it gives off a dorsal cutaneous branch. At the wrist, the nerve divides into superficial and deep branches with the deep branch traveling through Guyon’s canal [1]. The deep branch supplies the hypothenar muscles, the interossei muscles, and the third and fourth lumbricals. The superficial branch provides sensory branches to the ulnar one half of the fourth digit and the fifth digit. and motor branches to the palmar brevis muscle [2].

To our knowledge, the variant presentation of the ulnar nerve, as seen in the present case, has never been reported in the extant medical literature. The details of this unusual case and a review of the salient literature are therefore presented.

## Case presentation

**Figure 1:**
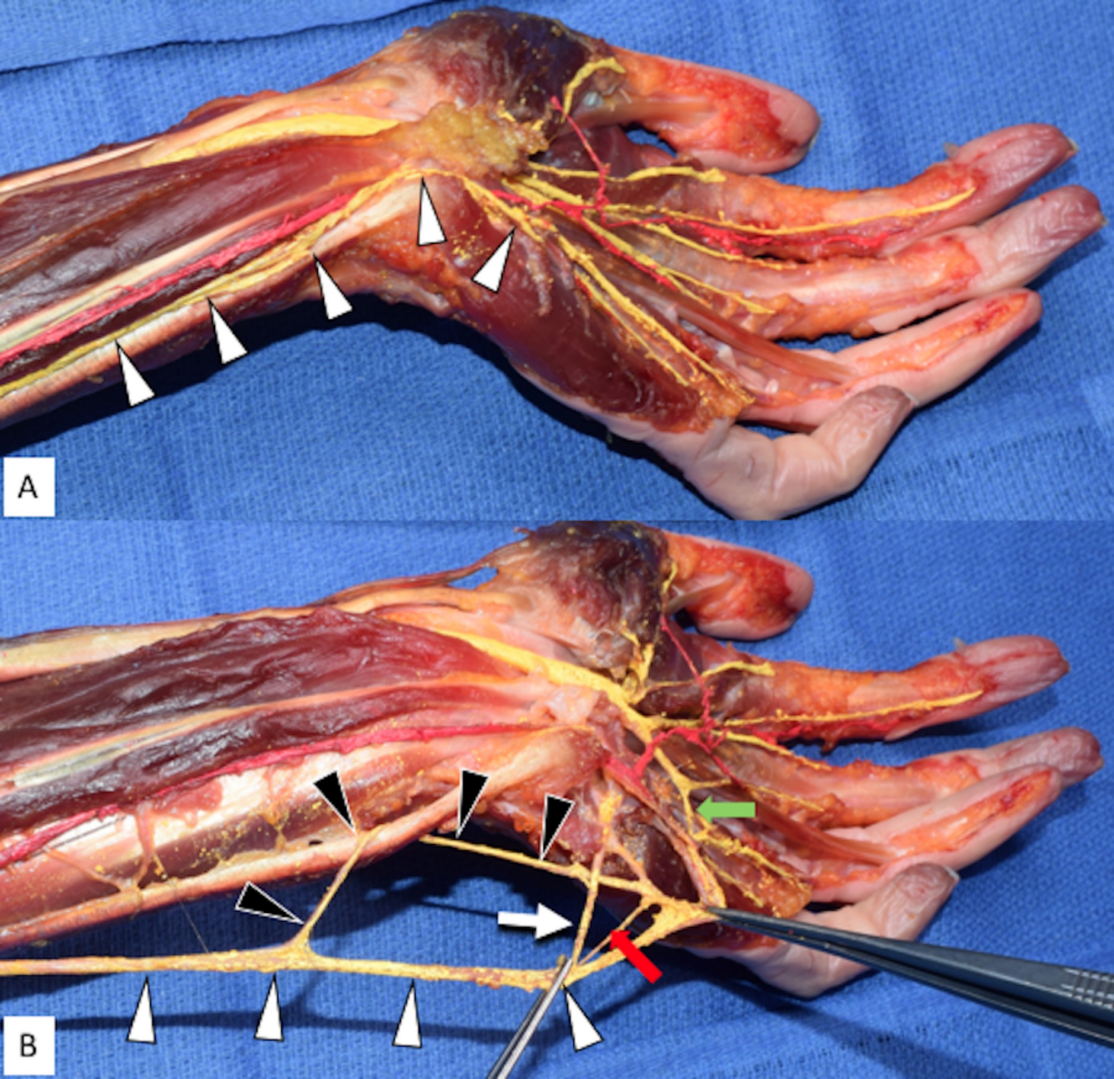
Left Anterior Forearm and Hand Dissection Figure 1A: Left palmar surface of the left hand and distal anterior forearm displaying the ulnar nerve in its anatomical position (arrowheads). Figure 1B: Figure 1A with the ulnar nerve main trunk rotated 180 degrees and then retracted medially. Note the variant loop formed around the flexor carpi ulnaris. White arrowheads: ulnar nerve main trunk; Black arrowheads: variant loop around the distal tendon of the flexor carpi ulnaris; White arrow: deep branch of the ulnar nerve; Superficial branch of the ulnar nerve retracted at right forceps; Red arrow: additional nervous interconnection; Green arrow: Riché-Cannieu anastomosis between the superficial ulnar nerve branch and median nerve in the hand.

During the routine dissection of the left forearm and hand in a fresh-frozen cadaver (aged 59 years at death), a variation of the distal ulnar nerve was observed. Along the lateral border of the flexor carpi ulnaris muscle, the ulnar nerve created a loop which traveled behind the muscle and lateral to the pisiform (Figures 1A, 1B). This dorsal branch of the ulnar nerve arose from the medial aspect of the loop (Figure 1B). The main trunk of the ulnar nerve distal to the origin of the dorsal branch gave rise to three branches in the hand distal to the pisiform bone. These branches were the superficial and deep ulnar nerve branches and an additional ulnar branch (Figure 1B). The superficial branch of the ulnar nerve was found to connect with the median nerve in the hand forming the so-called Riché-Cannieu anastomosis (Figure 1B). The deep branch of the ulnar nerve traveled to the interosseous muscles without providing a branch to the hypothenar muscles, which were innervated by an unknown branch of the ulnar nerve (Figure 1B). A palmar cutaneous branch of the ulnar nerve was not observed and no other anatomical anomalies were noted. No gross findings of previous surgical intervention, pathology, or trauma to the dissected region were identified. This variant of the ulnar nerve seen on the left side was not found on the right side of this cadaveric specimen.

## Discussion

A review of the extant medical literature failed to identify another similar description of the ulnar nerve variant as described herein. For example, a detailed review of ulnar nerve variations, as presented by Depukat et al. [3], did not describe a case such as ours. Although apparently rare, variants, such as the present case, should be known to those who treat patients with pathology or injury to the peripheral nerves [4-7].

The clinical presentation of an ulnar nerve neuropathy at the wrist depends on which of its branches are injured. A common injury site involving the ulnar nerve is at the hook (hamulus) of the hamate. Compression from a fractured hamate can injure the superficial or deep branches of the ulnar nerve [4]. However, due to the variant loop of the nerve, certain functions of the aforementioned nerves might remain intact. This is possible due to the anastomosis around the medial side of the pisiform with the hypothenar branch, additional branch, and superficial branch of the ulnar nerve. Additionally, lacerations or soft tissue swelling to this region of the wrist could cause uncommon presentations of the ulnar nerve neuropathy due to the anastomosing variant loop, as seen in the present case. 

The clinical presentations of the ulnar nerve at the wrist when normal anatomy is present are as follows:

1)   Damage to the dorsal cutaneous branch presents with sensory deficits to the dorsal surface of the fourth and fifth digits, sparing the distal ends and the dorsomedial surface of the hand [1]. 

2)   Damage to the superficial branch presents with motor deficits of the palmar brevis muscle used during gripping and with sensory deficits of the fourth (ulnar one-half) and fifth digits.

3)   Damage to the deep branch presents with atrophy of the hypothenar eminence and motor deficits resulting in a claw hand deformity [2].

## Conclusions

The variation of the ulnar nerve presented here appears to be unique. The exact function of such a derailed anatomy is not clear but, if found during surgery, might confound normal dissection methods or, when present, could result in varied clinical presentations regarding the sensory or motor examination of the hand.
